# Inverted U-Shaped Dose-Response Curve of the Anxiolytic Effect of Cannabidiol during Public Speaking in Real Life

**DOI:** 10.3389/fphar.2017.00259

**Published:** 2017-05-11

**Authors:** Antonio W. Zuardi, Natália P. Rodrigues, Angélica L. Silva, Sandra A. Bernardo, Jaime E. C. Hallak, Francisco S. Guimarães, José A. S. Crippa

**Affiliations:** ^1^Department of Neuroscience and Behavior, Ribeirão Preto Medical School, University of São PauloSão Paulo, Brazil; ^2^National Institute of Science and Technology for Translational Medicine, National Council for Scientific and Technological DevelopmentRio de Janeiro, Brazil; ^3^Department of Pharmacology, Ribeirão Preto Medical School, University of São PauloSão Paulo, Brazil

**Keywords:** cannabidiol, dose-response, anxiety, healthy volunteers, clonazepam, public speaking

## Abstract

The purpose of this study was to investigate whether the anxiolytic effect of cannabidiol (CBD) in humans follows the same pattern of an inverted U-shaped dose-effect curve observed in many animal studies. Sixty healthy subjects of both sexes aged between 18 and 35 years were randomly assigned to five groups that received placebo, clonazepam (1 mg), and CBD (100, 300, and 900 mg). The subjects were underwent a test of public speaking in a real situation (TPSRS) where each subject had to speak in front of a group formed by the remaining participants. Each subject completed the anxiety and sedation factors of the Visual Analog Mood Scale and had their blood pressure and heart rate recorded. These measures were obtained in five experimental sessions with 12 volunteers each. Each session had four steps at the following times (minutes) after administration of the drug/placebo, as time 0: -5 (baseline), 80 (pre-test), 153 (speech), and 216 (post-speech). Repeated-measures analyses of variance showed that the TPSRS increased the subjective measures of anxiety, heart rate, and blood pressure. Student-Newman-Keuls test comparisons among the groups in each phase showed significant attenuation in anxiety scores relative to the placebo group in the group treated with clonazepam during the speech phase, and in the clonazepam and CBD 300 mg groups in the post-speech phase. Clonazepam was more sedative than CBD 300 and 900 mg and induced a smaller increase in systolic and diastolic blood pressure than CBD 300 mg. The results confirmed that the acute administration of CBD induced anxiolytic effects with a dose-dependent inverted U-shaped curve in healthy subjects, since the subjective anxiety measures were reduced with CBD 300 mg, but not with CBD 100 and 900 mg, in the post-speech phase.

## Introduction

In just over half a century of research on cannabidiol (CBD) investigators described a broad range of pharmacological effects of the drug, many of which of therapeutic interest (Zuardi, 2008; Izzo et al., 2009). Among the possible therapeutic properties we can highlight CBD’s anxiolytic (Guimarães et al., 1990; Zuardi et al., 1993; Resstel et al., 2006; Crippa et al., 2010), antipsychotic (Zuardi et al., 1991; Moreira and Guimarães, 2005; Leweke et al., 2012), sleep-regulating (Chagas et al., 2014a), antidepressant (Zanelati et al., 2010), antiepileptic (Devinsky et al., 2015; Crippa et al., 2016), anti-inflammatory (Esposito et al., 2013), and analgesic (Boychuk et al., 2015) effects, in addition to improvement of Parkinson symptoms (Zuardi et al., 2009; Chagas et al., 2014a,b).

However, several animal studies have shown that CBD produces inverted U-shaped dose-response curves. These curves were first described in rats tested in the elevated plus maze (EPM) model of anxiety (Guimarães et al., 1990). In that study, CBD increased open arm exploration (an anxiolytic-like effect) at doses of 2.5–10 mg/kg, but not at 20 mg/Kg (Guimarães et al., 1990). Similar bell-shaped dose-response curves have been described in other studies investigating the behavioral effects of CBD. For example, zebrafish treated with 0.5 mg/kg CBD spent significantly more time in the upper zone of the aquarium (which is interpreted as an anxiolytic-like effect), while the behavior of fish treated with the lowest (0.1 mg/kg) and the highest doses (5 and 10 mg/kg) did not differ from controls (Nazario et al., 2015). A similar pattern was also observed in spontaneously hypertensive rats (SHRs), which present a schizophrenia-like behavioral phenotype. The prepulse inhibition in this strain of rats was reversed by 30 mg/kg of CBD, but not by the doses of 15 and 60 mg/kg (Levin et al., 2014). The same was observed using the model of reserpine-induced cognitive impairment in rats, an animal model of both Parkinson’s disease and tardive dyskinesia. Whereas CBD, at the dose of 0.5 mg/kg, attenuated the reserpine-induced memory deficit in the discriminative task, the dose of 5 mg/kg proved ineffective (Peres et al., 2016).

There is a clear need to explore new ways of managing anxiety disorders, since their treatment remains problematic and usually involves a combination of medications, including benzodiazepines and antidepressants. These drugs have their disadvantages such as risk of dependence and withdrawal syndrome, sexual side-effects, cognitive and psychomotor impairment, delayed onset of action (antidepressants), low acceptance, and requirement of careful dosage control (Katzman et al., 2014; Bandelow et al., 2015; Sgnaolin et al., 2016). CBD does not seem to induce significant adverse effects in humans (Bergamaschi et al., 2011b). However, from a possible therapeutic perspective, it is essential to determine if a similar inverted U-shaped dose-response pattern is also present in humans. In order to investigate this possibility, we chose one of the most consistent effects of CBD, the acute decrease in anxiety. This effect has been described in several animal models and in healthy volunteers and patients with social anxiety disorder (SAD) (Guimarães et al., 1990; Zuardi et al., 1993; Bergamaschi et al., 2011a; Crippa et al., 2011); however, it has not been investigated using multiple doses in humans.

One broadly used method for inducing experimental anxiety in humans is the Simulated Public Speaking Task (SPST – Graeff et al., 2003). The SPST is very consistent in elevating self-rated anxiety. However, its effects on physiological responses (heart rate, blood pressure, and cortisol secretion) are less consistent (Palma et al., 1994; Hetem et al., 1996; de-Paris et al., 2003). A meta-analysis of 11 studies that used the SPST confirmed this observation, showing a significant increase in overall effect size on subjective anxiety, but not on systolic blood pressure (SBP) and heart rate (Zuardi et al., 2013). Sparse evidence in the literature suggests that public speaking in a real-life situation is more efficient to increase physiological responses (Turner et al., 1990; Dickerson and Kemeny, 2004). In a recent study, we compared the test of public speaking in a real situation (TPSRS) and the SPST. The results showed that both methods were effective in increasing subjective anxiety, but only the TPSRS increased heart rate, SBP, and diastolic blood pressure (DBP) (Zuardi et al., 2013).

The present study, therefore, was designed to test the hypothesis that increasing doses of CBD would produce anxiolytic effects in an inverted U-shaped dose-response pattern in healthy volunteers submitted to the TPSRS.

## Materials and Methods

### Subjects

Sixty healthy men and women aged 18–35 years, with no history of past or current psychiatric illness, alcohol or other drug dependence were recruited through advertisement in the campus of the University of São Paulo in Ribeirão Preto. Participants with major medical conditions or who were taking medications with the potential to interfere with the study’s results were not included. The volunteers were interviewed in order to assess eligibility and to measure their propensity to anxiety through the trait version of the Spielberger State-Trait Anxiety Inventory (STAI- Spielberger et al., 1970). Eligible participants who agreed to participate were instructed to abstain from drugs in the week prior to the tests and from alcohol and caffeine in the 24 h before the test. All volunteers gave written informed consent to participate after being fully informed of the research procedures, which conformed to the current terms of the Declaration of Helsinki and were approved by the Ethics Committee of the Ribeirão Preto Medical School University Hospital (HCRP -No. 12407/09).

The volunteers were randomly allocated to five groups with 12 subjects each to receive different doses of CBD (100, 300, and 900 mg), clonazepam (1 mg) or placebo in a double-blind, randomized design. The groups were matched according to gender, age, body mass index (BMI), and STAI-trait score. One volunteer from the CBD-300 group could not attend the experimental session due to personal issues and was withdrawn from the study.

### Drugs

Cannabidiol powder with 99.6% purity (no other cannabinoids present) dissolved in corn oil at doses of 100 and 200 mg/ml was kindly provided by *Biosynthesis Pharma Group* (BSPG-Pharm, Sandwich, UK). The CBD solution, clonazepam tablets (Rivotril^^®^^, Roche Lab), and placebo (corn oil) were packed in identical gelatin capsules. All volunteers received five capsules of one milliliter each, in the following combinations: five capsules containing corn oil (placebo group); one capsule of CBD 100 mg/ml and four capsules of corn oil (CBD-100 group); three capsules of CBD 100 mg/ml and two capsules of corn oil (CBD-300 group); four capsules of CBD 200 mg/ml and one capsule of CBD 100 mg/ml (CBD-900 group); and one capsule with clonazepam and four capsules of corn oil (clonazepam group – CLON). Each treatment received a random number and both the investigator in charge of dispensing the capsules and the volunteer were unaware of their contents (double-blind). Finally, the choice of the interval between the drug administration and the TPSRS was based on previous pharmacological studies showing that the peak plasma concentrations of CBD and CLON taken orally usually occurs between 2 and 3 h after ingestion (Agurell et al., 1981; Crevoisier et al., 2003; Borgwardt et al., 2008; Fusar-Poli et al., 2009; Martin-Santos et al., 2012). CBD undergoes a significant first-pass effect leading to the formation of a number of metabolites and its half-life in humans was found to be between 2 and 5 days following oral administration. The bioavailability of oral CBD in humans is around 6%, thus supporting the view that it has a substantial first-pass effect (Zhornitsky and Potvin, 2012). The mean plasma level of CBD at 1, 2, and 3 h after the acute administration of 600 mg were 0.36, 1.62, and 3.4 ng/ml, respectively (Martin-Santos et al., 2012). The choice of the doses used in this study was based on previous evidence showing that the acute oral administration of 300 mg of CBD had anxiolytic effects in the SPST (Zuardi et al., 1993).

### Psychological Measurements

Two factors of the Visual Analog Mood Scale (VAMS), translated and validated into Portuguese by Zuardi and Karniol (1981), were used to evaluate anxiety levels and sedative effects during the test. The VAMS is a self-administered instrument consisting of 16 items. Prior to the TPSRS, each volunteer underwent a training session to complete the scale. The subjects were asked to mark the point that identified his/her current subjective state on a 100-mm straight line placed between two words that describe opposite mood states (e.g., relaxed-tense). A factorial analysis of the Portuguese version of the scale identified four factors (Zuardi et al., 1993): (1) ‘anxiety,’ comprising the items calm–excited, relaxed–tense, and tranquil–troubled; (2) ‘sedation,’ including the items alert–drowsy and attentive–dreamy; (3) ‘cognitive impairment,’ including quick-witted–mentally slow, proficient–incompetent, energetic–lethargic, clear-headed–muzzy, gregarious–withdrawn, well-coordinated–clumsy, and strong–feeble; and (4) ‘discomfort,’ including the items interested–bored, happy–sad, contented–discontented, and amicable–antagonistic (Parente et al., 2005). Since previous studies with both anxiogenic (Crippa et al., 2004; Parente et al., 2005) and anxiolytic drugs including CBD (Crippa et al., 2004, 2011; Bergamaschi et al., 2011a) have shown that only the anxiety and sedation factors present changes during public speaking tasks, we used only the items of these factors of the VAMS to reduce the administration time and allow the execution of the protocol. This procedure was used previously in the validation study of the PSRST (Zuardi et al., 2013).

### Physiological Measurements

Systolic blood pressure, DBP, and heart rate (HR) were measured with a digital sphygmomanometer (Omron, Brazil).

### Test of Public Speaking in a Real Situation (TPSRS)

In this procedure, each subject must speak in front of a group and, in the same experimental session, participate in the audience when the other members of the group are speaking. The audience is instructed to remain silent and with a neutral expression during the speeches.

Five experimental sessions were carried out with 12 subjects each (except 1, that included 11 subjects). At the beginning of the session, the volunteers were informed of the number of the treatment they would receive (as detailed above). The volunteers were then instructed to take seats marked with their respective treatment numbers. The seats were numbered in such a way that, at the end of the five sessions, the subjects of each treatment group had occupied all possible positions. The sequence of procedures always followed the same order from the first to the twelfth chair in an effort to minimize a possible effect of order in the sequence of speakers. For example, subjects in the CBD-300 group occupied the following positions: first session – 3, 8; second session – 4, 9; third session – 5, 10; fourth session – 1, 6, 11; fifth session – 2, 7, 12.

### Procedure

Volunteers began the experimental session after a minimum of 6 h of sleep and 2 h after eating a standard breakfast (200 ml chocolate milk and 100 g of bread with margarine).

The 12 volunteers sat in chairs arranged in a semicircle, following a predetermined order by the treatment number assigned to each one of them and performed the baseline measurements, followed by the intake of the five capsules within a 5-min interval for each volunteer. This phase was completed over a period of 1 h. The volunteers then watched a film about birds of Amazon forest lasting about 20 min. After that, the measurements were repeated with an interval of 5 min for each subject (pretest measure). 2 h and 20 min after the first volunteer had received the treatment, they all watched a video lasting about 10 min with recorded instructions for the speech test. 2 h and 30 min after the first volunteer received the treatment, they started performing the speech test, which lasted 5 min. Subsequently, the other volunteers were submitted to the speech test in the same order in which they had received the treatments. The speech test consisted of silently preparing a 1-min speech on a theme related to “the conditions of one public service of your city,” chosen by chance from 12 options and revealed immediately before the speech preparation. The prepared speech was then presented for 2 min, with the other participants and the investigators serving as the audience. The speech was interrupted halfway for the measurements. The sequence of the procedures in this phase was as follows: (a) seating on a chair arranged in front of others; (b) picking the speech theme; (c) thinking about the theme for 1 min; (d) starting the speech, which was interrupted after 1 min; (e) completing the self-assessment scale (VAMS anxiety and sedation factors) and physiological measurements; (f) continuing the speech for another minute. This sequence lasted for 5 min at most, such that, after 1 h, all volunteers had gone through the procedure. The final phase of the study started 216 min after the first volunteer had taken the drug. All the measurements were repeated, keeping the 5-min interval for each volunteer. **Table 1** presents an outline of the experimental procedure.

**Table 1:** Timetable of the experimental session.

Time (min)	Phase	Procedure	Measurement
-15		Adaptation to the laboratory	
0	Baseline (B)	Measurements and drug intake	VAMS-anxiety and sedation, blood pressure, heart rate
60		Film (about 20 min)	
80	Pre-stress (P)	Measurements	VAMS-anxiety and sedation, blood pressure, heart rate
140		Instructions about the PSRST	
150		Speech preparation	
152		Beginning of speech (1 min)	
153	Speech performance (S)	Measurements	VAMS-anxiety and sedation, blood pressure, heart rate
155		Continuation of speech (1 min)	
156		End of speech	
216	Post-stress (F)	Measurements	VAMS-anxiety and sedation, blood pressure, heart rate

*VAMS, Visual Analog Mood Scale.*

### Statistical Analysis

Clinical and demographic characteristics were analyzed with non-parametric tests (gender) and one-factor analysis of variance (ANOVA; age, BMI, and STAI-trait).

The scores in the two factors of the VAMS, arterial systolic and diastolic pressure, and heart rate were analyzed with a repeated-measures analysis of variance (repeated-measures ANOVA) including the factors ‘phase,’ ‘groups,’ and phase-group interaction. In cases where sphericity conditions were not reached, the degrees of freedom of the repeated factor were corrected with the Huynh-Feldt epsilon. Comparisons among the groups were made at each phase using a one-factor ANOVA followed by multiple comparisons with the Student-Newman-Keuls test.

Data analysis was performed using the Statistical Package for the Social Sciences (SPSS) version 17, and the significance level adopted was *p* < 0.05.

## Results

The demographic and clinical characteristics of the subjects are shown in **Table 2**. The groups did not differ significantly in respect to gender, age, BMI, and STAI-trait scores.

**Table 2:** Demographic and clinical characteristic of the groups.

	PLACEBO	CBD-100	CBD-300	CBD-900	CLON	χ^2^ or ANOVA (p)
Gender [M/F]	6/6	6/6	5/6	6/6	6/6	χ^2^= 0.07 (*p* = 0.99)
Age [mean (SD)]	22.0 (2.1)	22.5 (2.9)	22.6 (2.9)	23.3 (2.8)	22.1 (2.4)	*F*_4,57_ = 0.42 (*p* = 0.79)
BMI [mean (SD)]	23.2 (3.2)	22.4 (3.1)	23.3 (3.8)	24.0 (3.9)	22.9 (3.8)	*F*_4,56_ = 0.32 (*p* = 0.86)
STAI [mean (SD)]	41.4 (9.8)	46.5 (3.0)	46.4 (5.3)	46.0 (6.2)	41.1 (7.7)	*F*_4,54_ = 1.88 (*p* = 0.13)

**FIGURE 1:**
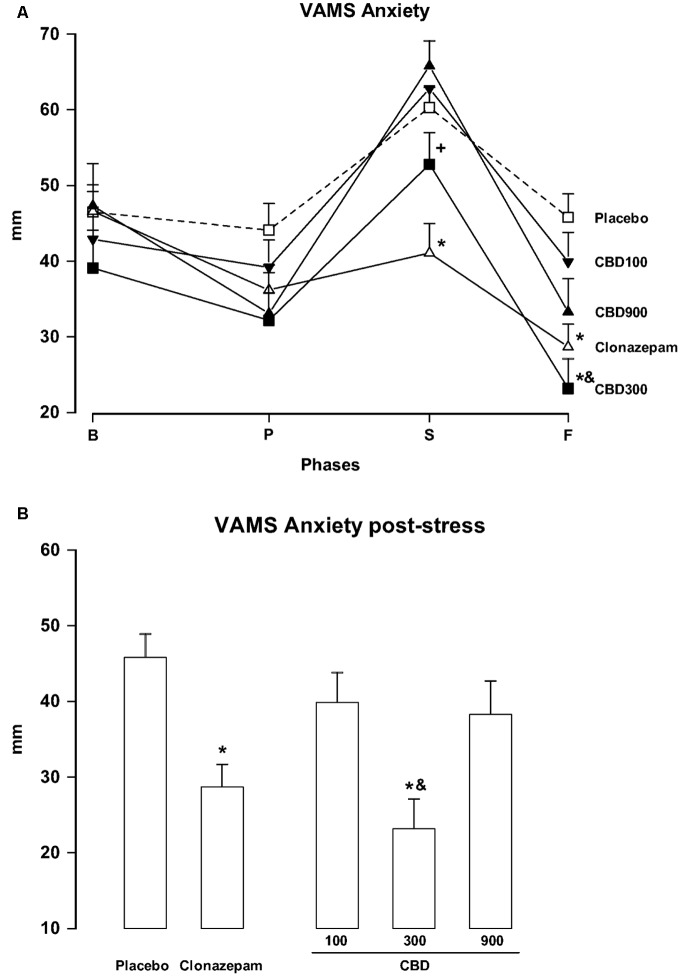
**Changes in the scores of the anxiety factor of the Visual Analog Mood Scale (VAMS) (mm) induced by public speaking in healthy volunteers (A)** during at baseline (B) and during the pre-stress (P), speech (S), and post-stress (F) phases **(A)**. For the sake of clarity, results from the last phase (F) are also shown as a bar graph **(B)**. Points represent the means ± SEM of 11–12 subjects. Asterisks (^∗^) indicate statistically significant differences compared to the placebo group, (+) indicates a significant difference compared to the CBD-900 group, and (&) indicates a significant difference compared to the CBD-100 group.

**FIGURE 2:**
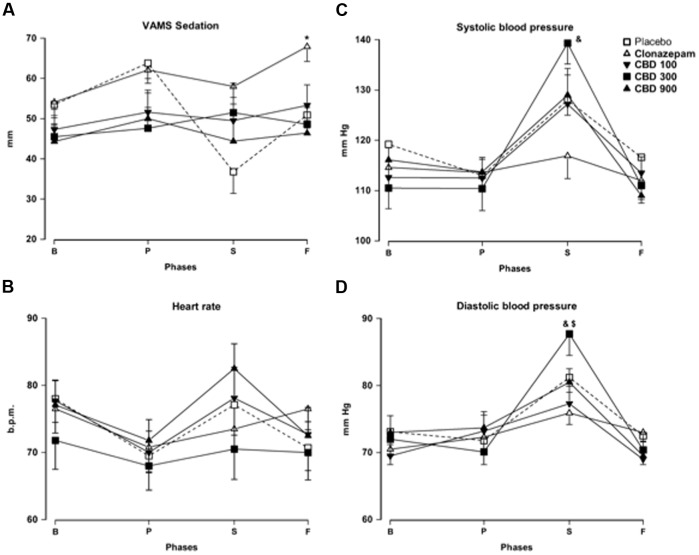
**Changes in the sedation factor of the VAMS (mm, A)**, heart rate [in beats per min (b.p.m.), **B**], systolic **(C)** and diastolic **(D)** blood pressure (mm Hg) induced by public speaking in healthy volunteers. Points represent the means ± SEM of 11–12 subjects. Asterisks (^∗^) indicate statistically significant differences compared to the other groups, (&) indicates a significant difference compared to clonazepam, and ($) indicates a significant difference compared to the CBD-100 group.

The repeated-measures ANOVA of scores in the anxiety factor of the VAMS showed a significant effect of phase (*F*_3,162_ = 60.24; *p* < 0.001), group (*F*_4,54_ = 3.25; *p* = 0.018), and phase-group interaction (*F*_12,162_ = 3.79; *p* < 0.001, **Figure 1A**). *Post hoc* comparisons among the groups in each phase showed significant differences (*p* < 0.05) between PLAC and CLON at the speech and post-stress phases. The scores of the CBD-300 group were significantly different from those of the CBD-900 group at the speech phase and from those of the PLAC and CBD-100 groups during the post-stress phase (*p* < 0.05). As an illustration, **Figure 1B** shows the inverted U-shaped dose-response curve induced by CBD at the post-stress phase. Regarding the sedation factor of the VAMS, the repeated-measures ANOVA showed significant effects of phase (*F*_2.16,116.7_ = 3.11; *p* = 0.028) and group (*F*_4,54_ = 3.48; *p* = 0.013, **Figure 2A**). A general *post hoc* comparison among the groups showed that clonazepam induced a higher sedative effect compared to the other groups (main effect of treatment). A similar effect was observed at the post-stress phase (*p* < 0.05).

The repeated-measures ANOVA showed significant effects of phase for the following physiological measures: systolic blood pressure (*F*_2.56,135.9_ = 71.00; *p* < 0.001), DBP (*F*_3,159_ = 43.39; *p* < 0.001), and heart rate (*F*_2.97,160,5_ = 10.02; *p* < 0.001), without differences among the groups (**Figures 2B–D**). The scores of these measures were high during the speech phase. The phase-group interaction was significant for systolic blood pressure (*F*_10.26,135.9_ = 5.64; *p* < 0.001) and DBP (*F*_12,159_= 3.24; *p* < 0.001). *Post hoc* comparisons among the groups in each phase showed significant differences between CLON and CBD-300 at the speech phase, both in systolic and DBP (*p* < 0.05). Significant differences in DBP were also found between the CBD-300 and CBD-100 groups (*p* < 0.05).

## Discussion

We used the TPSRS to experimentally induce anxiety and investigated whether the anxiolytic effect of CBD in humans produces an inverted U-shaped dose-response curve. In line previous evidence (Zuardi et al., 2013), this protocol significantly increased subjective anxiety, blood pressure, and heart rate, as shown by the significant effect of phase found in a repeated-measures ANOVA. Moreover, the TPSRS was sensitive in detecting the anxiolytic effect of clonazepam, a well-known anxiolytic drug, suggesting that it can be used to identify potentially anxiolytic drugs.

At the dose of 300 mg, CBD significantly decreased subjective anxiety compared to the PLAC group during the post-speech phase of the protocol. However, the same was not true for a lower (100 mg) or higher (900 mg) dose of the drug. Moreover, the subjective anxiety scores of the CBD-300 group also differed significantly from those of the CBD-900 group during the speech phase, and from the CBD-100 group during the post-speech phase. These results suggest that the acute anxiolytic effect of CBD followed an inverted U-shaped dose-response curve, which is consistent with results from animal studies (Guimarães et al., 1990; Levin et al., 2014; Nazario et al., 2015; Peres et al., 2016). Interestingly, the same dose of CBD that produced anxiolytic effects (300 mg) did not reduce systolic and DBP as clonazepam. This dissociation between anxiolytic and sympathoinhibitory effects following the acute administration of CBD is consistent with our observations using the SPST in healthy volunteers (Zuardi et al., 1993) and in patients with social phobia (Bergamaschi et al., 2011a).

The mechanism underlying this pattern of dose-response effect of CBD cannot yet be fully explained. In humans, limbic and paralimbic brain areas seem to be involved in the anxiolytic action of CBD. For instance, studies using single photon emission computed tomography (SPECT) in healthy volunteers and drug-naïve patients with SAD showed the involvement of the same areas implicated in the anxiolytic effect of CBD 400 mg, namely, the left parahippocampal gyrus and left amygdala-hippocampus complex (Crippa et al., 2004, 2011). Using functional magnetic resonance imaging (fMRI) in healthy volunteers, researchers found that CBD 600 mg attenuated activation in the amygdala and the anterior cingulate cortex during the recognition of fearful facial expressions (Fusar-Poli et al., 2009). The authors further explained that this action of CBD was mediated by alterations in subcortical prefrontal connectivity via amygdala and anterior cingulate cortex (Fusar-Poli et al., 2010).

Despite the identification of brain areas that may be involved in the anxiolytic effect of CBD, the specific pharmacological mechanism responsible for this effect remains to be elucidated. CBD is a drug with multiple pharmacological targets, including the interaction with several receptors (CB1, CB2, GPR55, TRPV1, and 5-HT1A), interference with the uptake and metabolism of endocannabinoids, and increase hippocampal neurogenesis in the adult brain (Zuardi et al., 2017). Several studies have supported the involvement of 5-HT1A receptors in the acute anxiolytic action of CBD. For example, the anxiolytic effect induced by systemic or intra-dorsal periaqueductal gray (DPAG) injections of CBD in rats is prevented by a 5-HT1A receptor antagonist (Campos and Guimarães, 2008; Soares et al., 2010). A similar interaction between CBD and 5-HT1A receptors in other brain structures related to the control of anxiety-like behaviors, such as the bed nucleus of the stria terminallis or the prelimbic frontal cortex, is also involved in the attenuation of anxiety responses (Gomes et al., 2011, 2012; Fogaça et al., 2014). In cultured cells, CBD has been reported to act as a 5-HT1A receptor agonist (Russo et al., 2005), although recent studies suggest that this is not the case in slices or *in vivo* conditions. The facilitation of 5-HT1A-mediated neurotransmission by CBD does not seem to involve blockade of 5-HT reuptake or changes in 5-HT1A mRNA expression in the DPAG after chronic CBD administration (Campos et al., 2013). These observations suggest that the modulation of 5-HT1A receptors by CBD is complex and could involve allosteric interactions (Rock et al., 2011).

As mentioned earlier, the mechanisms responsible for the inverted U-shaped dose-response curve of CBD are poorly understood. They could involve interactions with the vanilloid receptor 1 or TRPV1. At high concentrations, CBD can activate these receptors and facilitate glutamate release and defensive responses (Guimarães et al., 1991; Campos and Guimarães, 2009). This mechanism could mask CBD’s anxiolytic effect mediated by 5-HT1A receptors. Testing this possibility using intra-DPAG injections in rats, Campos and Guimarães (2009) showed that pretreatment with a TRPV1 antagonist (capsazepine) turned a higher and ineffective dose of CBD into an anxiolytic one. It remains to be tested if a similar mechanism is also involved in the U-shaped dose-response curve observed in our study. Consonant with previous evidence indicating that CBD has weaker sedative properties compared with benzodiazepines (Zuardi et al., 1993), the anxiolytic dose of CBD (300 mg) induced a significantly lower sedation level than clonazepam. It is an advantage that must be highlighted, since sedation and motor coordination impairment are among the most common adverse effects of benzodiazepines, in addition to potential dependence, cognitive deficits, and withdrawal symptoms, especially in the old age (Sgnaolin et al., 2016). These side-effects have not been observed with the use of CBD (Bergamaschi et al., 2011b).

The findings reported here need to be interpreted with caution, given the limitations of our study. First, it would have been desirable to measure the plasma levels of CBD and clonazepam and to relate these measurements to the magnitude of the anxiety levels; without a dose-response curve, doubts remain about whether the effects of CBD actually do follow an inverted U-shaped curve. It should be noted, however, that previous investigations have not been able to establish a direct relationship between plasma levels of CBD and its clinical effects (Agurell et al., 1986). Moreover, the small sample size limits the statistical power of the trial. Also, the study design does not allow us to rule out the possibility that another moderate dose (e.g., 400 or 600 mg) might have had even greater anxiolytic effects. For instance, previous studies have shown acute anxiolytic effects of 400 mg of CBD in SPECT studies in healthy volunteers (Crippa et al., 2004) and in patients with social phobia (Crippa et al., 2011) and of CBD 600 mg in an fMRI experiment with healthy men (Bhattacharyya et al., 2010). Although not necessary for this proof of concept study, these limitations could be overcome in future studies examining another 2–3 doses between 300 and 900 mg to establish a true therapeutic index.

## Conclusion

Our results are consonant with evidence from preclinical studies and support the view that CBD induces acute anxiolytic effects with an inverted U-shaped dose-response curve in humans. These findings stress the importance of the careful choice of dose ranges when investigating the potential therapeutic effects of CBD. Further studies assessing the dose-response curve of CBD in other conditions such as schizophrenia, pain, epilepsy, and Parkinson’s disease and involving the chronic administration of CBD are necessary to translate preclinical evidences into clinical practice and to determinate the precise therapeutic window of CBD for each condition.

## Author Contributions

AZ, JH, and JC designed the study. NR, AS, and SB conducted the experiments. AZ and FG conducted the statistical analysis. AZ wrote the first draft of the manuscript. All authors contributed to and had approved the final manuscript.

## Conflict of Interest Statement

AZ, JH, FG, and JC are co-inventors (Mechoulam R, JC, Guimaraes FS, AZ, JH, Breuer A) of the patent “Fluorinated CBD compounds, compositions and uses thereof. Pub. No.: WO/2014/108899. International Application No.: PCT/IL2014/050023” Def. US no. Reg. 62193296; 29/07/2015; INPI on 19/08/2015 (BR1120150164927). The University of São Paulo has licensed the patent to Phytecs Pharm (USP Resolution No. 15.1.130002.1.1). The University of São Paulo has an agreement with Prati-Donaduzzi (Toledo, Brazil) to “develop a pharmaceutical product containing synthetic cannabidiol and prove its safety and therapeutic efficacy in the treatment of epilepsy, schizophrenia, Parkinson’s disease, and anxiety disorders.” JH and JC have received travel support from BSPG-Pharm. The other authors declare that the research was conducted in the absence of any commercial or financial relationships that could be construed as a potential conflict of interest. The reviewers RWG, KA and handling Editor declared their shared affiliation, and the handling Editor states that the process nevertheless met the standards of a fair and objective review.
